# Identification of a large homozygous *SPG21* deletion in a Chinese patient with Mast syndrome

**DOI:** 10.1111/cns.13723

**Published:** 2021-09-07

**Authors:** Yan‐Yan Xue, Xue‐Rong Huang, Hai‐Lin Dong, Zhi‐Ying Wu, Hong‐Fu Li

**Affiliations:** ^1^ Department of Neurology and Research Center of Neurology in Second Affiliated Hospital, and Key Laboratory of Medical Neurobiology of Zhejiang Province Zhejiang University School of Medicine Hangzhou China; ^2^ Department of Neurology Ruian City People's Hospital Ruian China

## Abstract

A 37‐year old man presented a slight delay in early developmental milestones, cognitive decline, difficulty walking, cerebellar signs and extrapyramidal signs. Brain magnetic resonance imaging (MRI) showed a thin corpus callosum, cerebral atrophy, non‐specific white‐matter hyperintensity, and cerebellar atrophy. The genetic test revealed a putative homozygous deletion in *SPG21* from exon 3 through exon 7, which was further validated by long‐range primer‐walking PCR. This is the first report of Chinese patient with Mast syndrome carrying a large homozygous *SPG21* deletion.

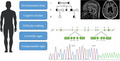

Dear Editors,

Mast syndrome (OMIM #248900) is an extremely rare autosomal recessive disease characterized by progressive hereditary spastic paraplegia (HSP), dementia, and other central nervous system symptoms. This disease was firstly described and occurred with high frequency in the Old Order Amish owing to an ancestral founder mutation in *SPG21* gene.^1^ *SPG21* (also known as *ACP33*) was identified as the causative gene of Mast syndrome in 2003.^1^ As the sole causative gene of Mast syndrome so far, there were only three variants in *SPG21* reported.^2^, ^3^ In this study, we reported the first Chinese patient with Mast syndrome carrying a large homozygous *SPG21* deletion.

The proband was a 37‐year old man born at term after an uneventful pregnancy with no family history of neurologic disease. A slight delay in early developmental milestone was noted as he started walking at around 17 months. Also, learn difficulties, bradyphrasia, and gait dysfunction were noted during the childhood. At the age of 8, he started wearing eyeglasses owing to hyperopia. At the age of 34, the patient presented progressive slow reaction, dysarthria, unsteady walking, and declined mental function with executive dysfunction. Additionally, he occasionally showed fecal incontinence since the age of 36. Notably, his parents were in consanguineous marriages (Figure 1A). The physical examinations revealed slow response to verbal communication, cerebellar ataxia, positive Babinski sign, and hyperreflexia in the lower limbs. Muscle strength and tension was normal. The patient had a high school degree. Mini‐mental state examination (MMSE) scored 25, Scale for the Assessment and Rating of Ataxia (SARA) scored 8, and International Cooperative Ataxia Rating Scale (ICARS) scored 20. Laboratory findings showed elevated triglyceride concentration and uric acid but normal liver transaminases. Other laboratory tests were unremarkable. Brain magnetic resonance imaging (MRI) showed a thin corpus callosum, cerebral atrophy predominantly in frontal lobe, non‐specific periventricular white‐matter hyperintensity, ventricle enlargement, and cerebellar atrophy (Figure 1B). Nerve conduction velocity revealed decreased sensory amplitude and velocity in his right sural nerve. Electromyogram showed no fibrillation potential or positive sharp waves in the muscle of limbs. Over a 6‐year follow‐up, his walking difficulties and cognitive impairments were deteriorated. In the distal lower limbs, muscle strength was 4/5, and muscle hypertonia was detected. MMSE scored 21, and Montreal Cognitive Assessment (MOCA) scored 11 (lower scores in visuospatial/executive function, attention, delayed recall, and orientation).

**FIGURE 1:**
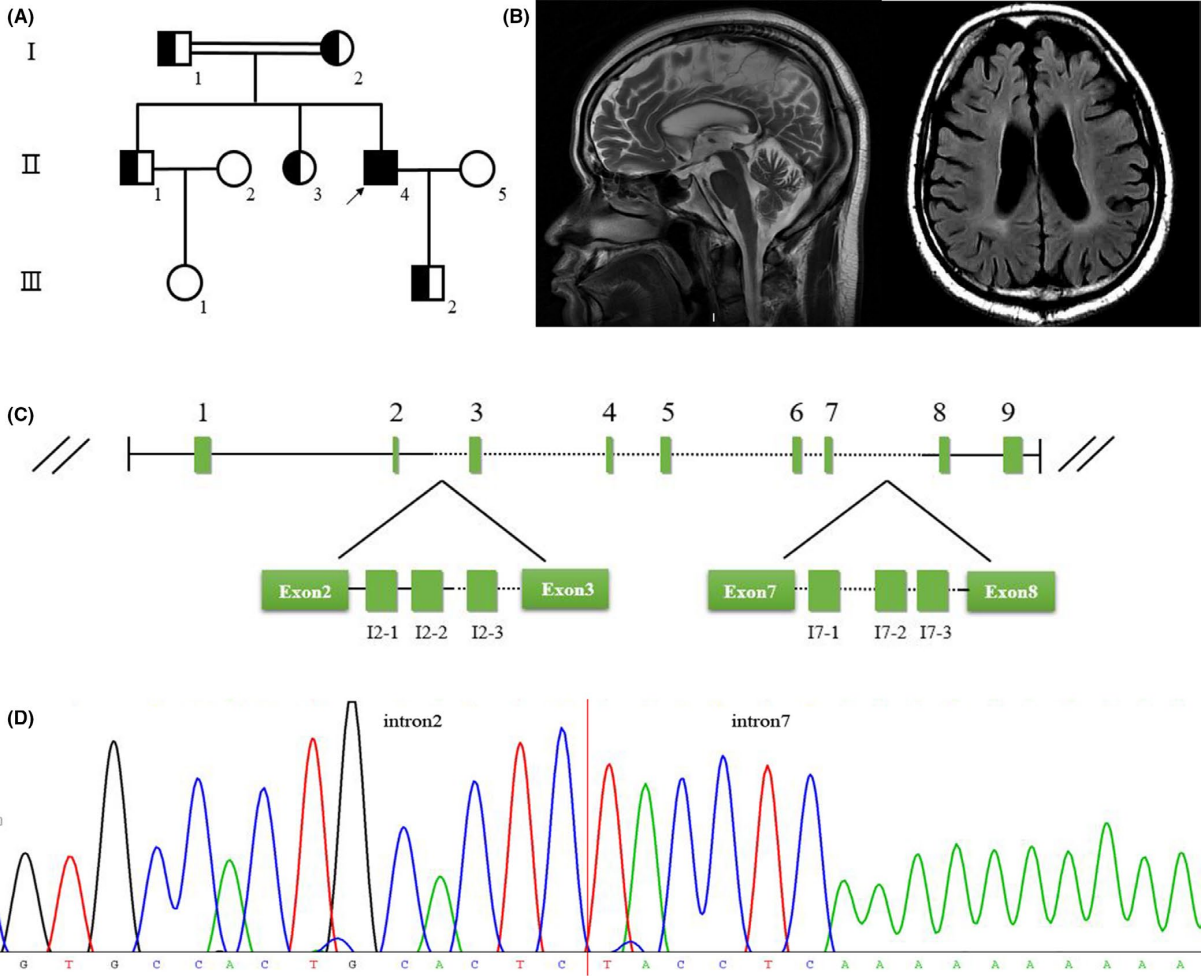
A Chinese family with a large homozygous SPG21 deletion. (A) Pedigree tree of the family; (B) proband brain MRI at the age of 37: sagittal T2‐weighted image (left) and axial T2 FLAIR image (right); (C) breakpoint detection of the large deletion of *SPG21* by primer‐walking PCR strategies. (D) Identification of the breakpoints by sequencing of the long‐range PCR product

Whole‐exome sequencing showed no pathogenic variants relevant to the above symptoms. However, analysis with ExomeDepth revealed a putative homozygous deletion in *SPG21* from exon 3 through exon 7. The deletion breakpoints (chr15: 64,965,751–64,981,338) and segregation were subsequently confirmed by long‐range primer‐walking PCR as shown in Figure 1C, D (details in supplemental methods and Table S1). Quantitative real‐time PCR targeting *SPG21* exon 8–9 region revealed unchanged mRNA expression in I‐1 and II‐4, compared with the normal familial member III‐1 (Figure S1). Therefore, this large deletion did not lead to nonsense‐mediated mRNA decay but produced a putative protein lacking 204 amino acid residues (p. (Ile22_Val224del)).

Mast syndrome is a complicated form of HSP and was rarely reported worldwide. It is caused by mutations within *SPG21* encoding a protein named “Maspardin,” which contained a noncatalytic alpha/beta hydrolase fold domain and was involved in the negative regulation of CD4 activity via protein‐protein interaction.^4^ Within the interaction region, Ser109 is critical for the interaction of maspardin with CD4. In addition, maspardin could interact with Rab7 GTPase, which orchestrate vesicular trafficking, maturation, and fusion.^5^ Although the specific mechanism was not clear, knockout of *SPG21* resulted in hind limb dysfunction in mice and increasing axon branching in neurons.^6^

Until now, only three variants in *SPG21* have been documented. Among these reported variants, two variants producing frameshifts (p. Arg40Glufs*27 and p. Thr201Asnfs*13) cause generally identical symptoms.^1^, ^3^ However, the Japanese patients carrying the homozygous missense variant (p. Ala108Pro), which was next to the critical site of the alpha/beta hydrolase fold domain (Ser109), showed strikingly late‐onset HSP symptoms without bulbar, extrapyramidal, or cerebellar signs.^2^ The large deletion in *SPG21* have not been reported before. In our study, the patient carrying large homozygous deletion of *SPG21* shared the most symptoms with patients carrying *SPG21* frameshift variants, but presented childhood‐onset hyperopia and early‐onset fecal incontinence. Hyperopia may be caused by other factors rather than *SPG21* mutation, given that his sister, a heterozygous mutation carrier, also had hyperopia since childhood. We supposed his fecal incontinence might result from frontal involvement, which is consistent with the imaging findings. To uncover the genotype‐phenotype correlation, the accumulations of patients with *SPG21* mutation are required. Recently, functional MRI (fMRI) has been performed in various central nervous system disease and revealed altered functional connectivity in different brain areas.^7^, ^8^ Conventional brain MRI usually reveals the structure alterations in complicated HSP, while fMRI could detect the alterations of the brain activities in both pure and complicated HSP, which emphasize the need for combined analysis of fMRI and structure MRI at multiple scales in Mast syndrome patients.^9^, ^10^

In addition, we also found this large deletion did not influence the level of *SPG21* mRNA, indicating the disappearance of the allele mainly causes the dysfunction of corresponding protein. Due to the technical and material limitations, we did not perform functional study for the mutant protein, which is one limitation of our study.

In conclusion, we report the first Chinese patient with Mast syndrome carrying a large homozygous *SPG21* deletion. Our study broadens the genetic and phenotypic spectrums of Mast syndrome.

## CONFLICT OF INTEREST

The authors have no competing interests to declare.

## CONSENT TO PARTICIPATE

The patient provided written consent for participation.

## CONSENT FOR PUBLICATION

The patient provided written consent for disclosure of medical information and images.

## Supporting information

Supplementary Material

## Data Availability

Data will be available upon reasonable request.
