# Supplementary material for: Identification of a large homozygous SPG21 deletion in a Chinese patient with Mast syndrome
Source: CNS Neurosci Ther. 2021 Sep 7;27(10):1251–3. doi: 10.1111/cns.13723 (PMC8446208; doi:10.1111/cns.13723)
Supplement: Supplementary file 1 — Supplementary Material [file CNS-27-1251-s001.docx]

SUPPLEMENTAL MATERIAL

**Identification of a large homozygous SPG21 deletion in a Chinese patient with Mast syndrome**

1. **Supplemental methods**

**Genetic analysis**

Genomic DNA was extracted from peripheral blood leukocytes using the QIAamp® DNA Blood Mini Kit (QIAGEN, Germany). The prepared DNA sample of the patient was captured by the Agilent Sure Select Human All Exon V6 products and sequenced on the Illumina HiSeq X Ten platform (XY Biotechnology Co. Ltd., Hangzhou, China). The CNV analysis of the Whole-exome sequencing (WES) data was performed using standard parameters for ExomeDepth software.

**The breakpoint analysis of the large deletion of *SPG21***

As the CNV analysis of WES data identified the homozygous deletion from intron 2 through exon 7 in the *SPG21*, primer-walking polymerase chain reaction (PCR) was performed to localize the breakpoints between intron 2 and intron 7. Firstly, the primer walking strategy was applied to narrow down the region of breakpoints with 3 fragments (I2–1 to I2-3) located in intron 2 and 3 fragments (I7–1 to I7–3) in intron 7 (primers sequences shown in Table S1). Long-range PCR was subsequently applied to precisely identify the breakpoints with the KOD DNA Polymerase (Toyobo, Japan) (primers sequences shown in Table S1). The PCR products were analyzed by electrophoresis and sequenced.

| **Primer** | **Sequence (5’- 3’)** |
| --- | --- |
| Primer walking detection of *SPG21* | |
| SPG21-I2-1F | GTTCACTCCTTCCCTGTCATGT |
| SPG21-I2-1R | GCAGAGTCAATACTCAACTGCAA |
| SPG21-I2-2F | GCTGATTCCCTTGCAGGAGT |
| SPG21-I2-2R | AAGGCAAAGTCCAGAGAGGC |
| SPG21-I2-3F | TCTGGACTTTGCCTTGCTCC |
| SPG21-I2-3R | GGCTGCTGCTTCTCTGTTTC |
| SPG21-I7-1F | CTTTCTGGCAGGAATAACACCAT |
| SPG21-I7-1R | TGTTACCCTTGTGAAGCACCTT |
| SPG21-I7-2F | TTGCCTTATAAATGGATTTGTTCCT |
| SPG21-I7-2R | TGGCAAATGTCAGAATGCAGC |
| SPG21-I7-3F | ACAGGACATTTGCTCTGCCT |
| SPG21-I7-3R | TGCACAGCATCTCCTGCATT |
| LR-PCR amplification | |
| F | CAGCTACTTGGGAGGCTGAG |
| R | AGCAGCCTGAACTACCTCCA |
| quantitative real-time PCR | |
| F | GTGCAGAAGTGCAGAGGTCA |
| R | TACTGCTCCTCCTGGCTGAT |

1. **Supplemental Table**

**Table S1. Primer sequences in this study.**

1. **Supplemental Figure**


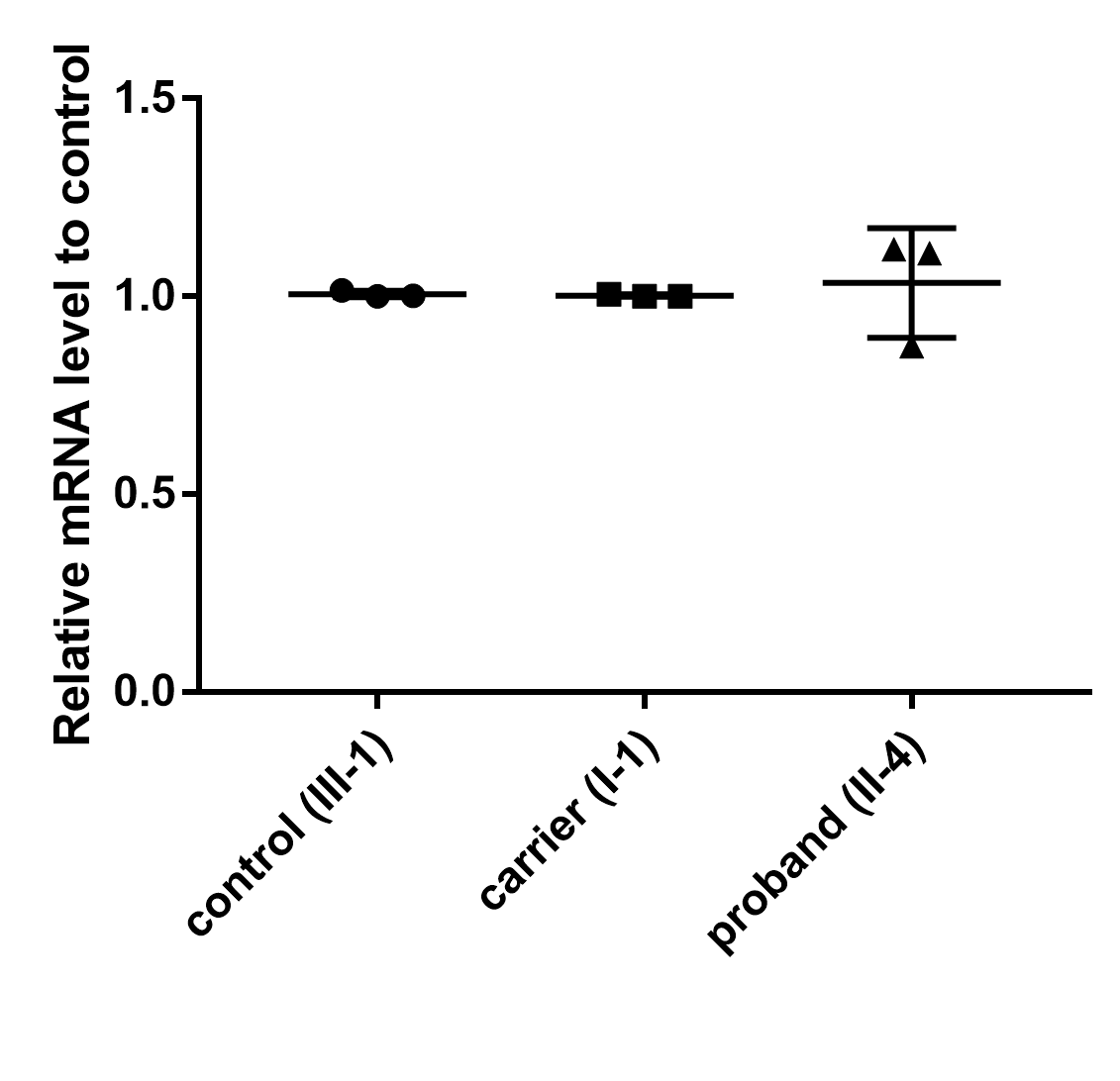


**Fig.S1 Quantitative real-time PCR revealed unchanged *SPG21* gene expression levels in the patient.**
